# Porcine/Chicken or Human Nephropathy as the Result of Joint Mycotoxins Interaction 

**DOI:** 10.3390/toxins5091503

**Published:** 2013-09-04

**Authors:** Stoycho D. Stoev, Stefan A. Denev

**Affiliations:** 1 Department of General and Clinical Pathology of Faculty of Veterinary Medicine, Trakia University, Students campus, 6000 Stara Zagora, Bulgaria; 2 Department of Biochemistry and Microbiology of Faculty of Agriculture, Trakia University, Students campus, 6000 Stara Zagora, Bulgaria; E-Mail: stefandenev@hotmail.com

**Keywords:** mycotoxic porcine nephropathy, mycotoxic chick nephropathy, Balkan endemic nephropathy, joint mycotoxin effect, mycotoxin interaction, ochratoxin A, penicillic acid, fumonisin B_1_, citrinin, unknown metabolite

## Abstract

A survey was made of the literature concerning the occurrence and incidence of mycotoxic nephropathy in pigs and chicks in different countries. Various etiological factors contributing to the development of the disease were considered. The main nephrotoxic fungi as well as the specific conditions for their growth and toxins production were briefly described. A survey was made about the most frequent nephrotoxic fungal contaminants in various feedstuffs from plant origin. In addition, their natural quantities and importance for development of mycotoxic porcine/chick nephropathy (MPN/MCN) are also explored. In addition, a survey was made of the feedstuffs representing the most favorable environment for nephrotoxic fungal growth as well as the most favorable storehouse conditions for this fungal growth were shortly described. The significance of some underestimated fungal species, which can provoke kidney damage, was studied. The importance of joint mycotoxin interaction and newly identified fungal metabolites in the complex etiology of mycotoxic nephropathy ranged in some countries is deeply investigated. The toxicity of the low contamination levels of some combinations of mycotoxins often administered by pigs and chicks in the practice was carefully studied.

## 1. Introduction

Mycotoxic nephropathy (MN), which is widely encountered renal disease all over the world, is usually a renal disorder caused by alimentary ingestion of some nephrotoxic mycotoxins. Ochratoxin A (OTA) is considered to be the most important mycotoxin provoking this nephropathy. However, this nephropathy was recently found to have much complicated pathology and etiology in some countries such as Bulgaria [1] and South Africa [2]. In these countries, spontaneous nephropathy in pigs [3,4,5] or chicks [6] described previously, was recently found to be provoked by several mycotoxins as OTA, penicillic acid (PA) and fumonisin B_1_ (FB_1_), having a synergistic interaction [1,2]. Some of the same mycotoxins (mainly OTA and PA) produced by “storage fungi” (such as *Penicillium* and *Aspergillus* species) are secondary fungal metabolites encountered in feedstuffs/foods kept in storehouse conditions and increased humidity. The other target mycotoxin FB_1_ is usually produced by “field fungi” mainly contaminated maize before harvesting [7]. 

The disease has obtained a great popularity in some countries with well-developed stock-breeding (especially pig-breeding) as Scandinavian countries. There are, however, some variances with the manifestation of this disease, especially with the clinicomorphological picture, which in many cases is influenced by the secondary bacterial infections as a result of the pronounced immunosuppression in the affected animals or by different combination of mycotoxins provoking this nephropathy. Therefore, this review paper could contribute to a better understanding of various differences in clinicomorphological findings in animals/chicks or humans suffering from spontaneous cases of MN and to timely disagnosis. The main focus will be, however, on clarifying the real etiological nature of various spontaneous cases of this nephropathy in different countries having different pathological manifestations. 

## 2. A Known Etiology of Classic Mycotoxic Nephropathy as Described for the First Time in Denmark

So far, only OTA and partly citrinin were causally associated with spontaneous cases of MN. Etiologic studies of a culture of *P*. *viridicatum Westling*, isolated from a nephrotoxic batch of barley [8], revealed the strain to be a producer of three nephrotoxic compounds: oxalate, citrinin (CIT) and OTA. Toxicologic studies of oxalate administered orally to pigs indicated that even an extremely high dose of sodium oxalate (1 g/kg b.w.) was only able to cause a slight interstitial fibrosis and no tubular damage [9]. Thus, oxalates were ruled out as causal determinants of nephropathy in pigs. In order to determine the significance of the two other compounds (CIT and OTA) in the porcine nephropathy, a survey was carried out in three districts of Denmark with high frequency of the disease for OTA and citrinin contamination of feeding forages. OTA has been found in a high proportion of the samples, whereas CIT has been a rarer contaminant. About 50% of the contaminated samples from the nephropathy districts contained more than 200 ppb OTA, which is the minimal level able to induce some nephropathy damages during the fattening period of bacon pigs, if it is ingested for about four months [10]. The highest concentration of CIT has been 2 ppm (or 2000 ppb), which is about 1% of the concentration required for development of nephropathy [9]. This seems to indicate that OTA is a major disease determinant of mycotoxic porcine nephropathy (MPN) in Denmark. OTA has proved to be nephrotoxic in all the species of mammals tested so far as well as in poultry. Therefore, based on many subsequent spontaneous and experimental studies, OTA was considered to be a primary cause of porcine/chicken nephropathy, a disease commonly recognized in Denmark and Sweden [11] as well as in many other countries in the world.

Ochratoxins present a group of structurally related compounds. However, the most toxic compound among this group is OTA, which was isolated first from *Aspergillus ochraceus* and chemically defined in 1965 by several South African scientists [12,13,14,15]. Recently, many new isolates of *Aspergillus* or *Penicillium* were found to produce OTA [1,2,16,17,18]. However, the main producers of OTA in field conditions usually are the strains *A*. *ochraceus* and *P*. *viridicatum*. Fungi that produce OTA are widespread in nature and commonly contaminate stored grain. The examination of naturally contaminated feedstuffs revealed that *Penicillium* genera (mainly *P*. *viridicatum* and *P*. *palitans*) were the main OTA and citrinin producing fungi in the temperate climatic areas of the world. These fungi usually develops on grains at low temperatures and are able to produce a considerable amount of the mentioned above mycotoxins even at 5 °C, a temperature commonly encountered during the winter season in colder climatic zones, like Northern Europe and Scandinavia, or at higher temperature up to 31 °C [19]. On the other hand, *Aspergillus ochraceus* probably is the most important producer of OTA in tropical and semitropical areas [20,21]. However, the growth of *A*. *ochraceus* on natural substrates has not been associated with a natural occurrence of OTA. Not all strains of *P*. *viridicatum* and *A*. *ochraceus* are OTA producers, but certain isolates produce other toxic compounds, e.g., citrinin, penicillic acid, some unknown metabolites (UM), *etc* [1,2,3,22,23].

It is known that OTA is primarily a contaminant of feed grains and cereals, especially maize, oats, wheat and barley. It is estimated that in Europe, at least 50% of the dietary OTA intake comes from cereals and cereal products [24]. It is found that barley, oats, wheat and maize grown in Scandinavian, India and Balkan countries are particularly susceptible to OTA contamination [1,2,25,26,27]. Animal feedstuffs from Europe, Canada and Australia may also be highly contaminated with OTA, sometimes concentrations exceeding 5000 ppb [25,28]. OTA is also frequently found in the soybeans, beans and peas [29,30], coffee-bean and roasted coffee [31,32,33,34,35,36], cocoa bean [29], wine and grape-juice [18,37,38,39,40,41], beer [42], various beverages including wines, beer, fruit juices [43], raisins [29], spices and herbs [44,45], and animal products [30]. Provisional estimates of the Codex Alimentarius Commission, based on limited European data, suggest that red wine is the second major source of human exposure to OTA following cereals and preceding coffee and beer [46]. The OTA concentration is also remarkably higher in red wines (22–1153 ng/L) than in white wine (12–108 ng/L) [47].

Various studies revealed that the kind of substrate on which *Penicillium* and *Aspergillus* species were grown greatly influenced OTA production. It is found that the oilseeds (peanuts and soybeans) supported a much higher production of OTA by *A*. *alutaceus* (or *A*. *ochraceus*) than the grains (maize and wheat), whereas the grains were a much better substrate for the production of OTA by *P*. *verrucosum* than the oilseeds [48].

Some profound investigations of the two major OTA producing species on agar media showed that the temperature range for growth of *A*. *ochraceus* and *P*. *viridicatum* is 8–37 °C and 0–31 °C respectively, whereas the temperature range for OTA production by *A*. *ochraceus* is 12–37 °C with an optimum yield between 31 and 37 °C and the range for toxin production by *P*. *viridicatum* is 4–31 °C with maximum OTA accumulation between 16 and 24 °C [21,49].

Investigation of ochratoxin production on sterile grain substrates demonstrated a maximum OTA accumulation at 25–28 °C during 7 to 14 days of fermentation [50,51]. Incubation beyond the period of maximum OTA accumulation may result in some decreases in toxin levels [52]. OTA is fairly stable, but some loss can occur with time under storage conditions.

The ochratoxin-producing species generally are found on grain stored at moisture above 15% [53]. The water content needed for OTA production by *P*. *viridicatum* was found to be between 18.5% and 21.6% [53]. The minimum temperature and water content in grains needed for OTA production by *P*. *viridicatum* are, respectively, 4 °C and 18.5% [53].

## 3. The Joint Mycotoxin Interaction and the Complex Etiology of Mycotoxic Nephropathy

It is important to mention that there are some variances with the clinicomorphological picture of that disease, which in many cases is provoking by combined nephrotoxic effect of several mycotoxins [1,2,22,23,54,55] (Table 1) and is additionally influenced by some secondary bacterial infections as a result of the significant immunosuppression in the affected animals [56,57,58,59,60]. 

**Table 1:** Possible mycotoxins involved in mycotoxic nephropathy in pigs/chick or humans in the Balkans often found as contaminants of feedstuffs/foods from endemic areas [1].

Mycotoxins	Producing species	Reference
OTA (ochratoxin A)	*P*. *viridicatum*, *P*. *Commune*, *A*. *niger*, *A*. *ochraceus*, *A*. *wentii*, * A*. *fumigatus* and others	[1]
PA (penicillic acid)	*P*. *polonicum*, *P*. *aurantiogriseum*, *P*. *viridicatum*, *P*. *Crustosum*, *A*. *ochraceus* and others	[1]
FB1 (fumonisin B1)	*Gibberella fujikuroi*, *F*. *verticillioides*	[1]
CIT (citrinin)	*P*. *citrinum*, *P*. *viridicatum*, * P*. *expansum*, *A*. *candidus and others*	[1]
UM (unknown metabolite)	*P*. *polonicum*, *P*. *aurantiogriseum*, *P*. *crustosum*, *P*. *viridicatum*, *P*. *chrysogenum*, *P*. *commune*, *P*. *citrinum*, *P*. *expansum*, *A*. *fumigatus*, *A*. *flavus*, *A*. *candidus* and others	[1]

**Figure 1:**
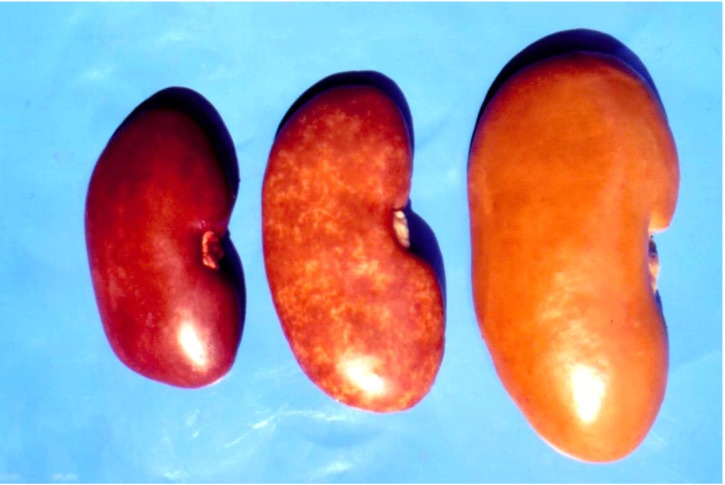
Range of enlargement and mottled or pale appearance of affected pig kidneys from spontaneous cases of mycotoxic porcine nephropathy (MPN) taken at slaughter time in Bulgaria.

OTA, CIT and FB_1_ are known nephrotoxic mycotoxins and probably all of them are involved in some cases of nephropathy in various countries [1,2,22,23,61,62,63]. Fumonisins are mycotoxins produced *by Fusarium verticillioides* (*F*. *moniliforme*), *Fusarium proliferatum* and related *Fusarium* species found mainly in corn (maize) [64]. On the other hand, penicillic acid (PA) can be produced by *P*. *aurantiogriseum* strains and the same ochratoxinogenic fungi from the *Aspergillus ochraceus* group, which are the major producers of OTA in the warmer climatic zones [3,22,23]. However, only scarce data are available about a combined exposure to OTA, CIT, FB1 and PA, which might spontaneously occur under field conditions [1,2] (Table 1). Recently, it was found that the contamination levels of OTA in Bulgarian and South African feedstuffs [1,2] from farms with nephropathies (100–200 µg/kg) were at least 5-fold less than that presented as the explanation for Danish mycotoxic porcine (MPN) or chick (MCN) nephropathy [3,4,6]. It seems that the animals’ intake of OTA is much lower than that shown to be necessary [65], even for less severe symptomatology, as can be seen from all experimental studies with OTA. The analysis for OTA of various feed samples from farms with high frequency of spontaneous MPN in Bulgaria and South Africa revealed that the values were substantially low and ranged from 38 to 552 ng/g (mean 207.1 ± 65.14 in Bulgarian farms with MPN) for year 1993 and from 42 to 427 ng/g (mean 114.06 ± 35.79 in Bulgarian farms with MPN) for year 1994 respectively [3]. Our recent studies fully support these findings and OTA was found in mean levels of 188.8 ± 27.3 ng/g for year 2006 in Bulgarian farms with MPN and 67.8 ± 39.2 ng/g for years 2007 or 75.2 ± 20.6 ng/g for years 2008 in South African farms with MPN [1,2]. These farms usually had a history of incorrect feed storage. Sometimes the problem seemed to come from certain feed plants whose grains, collected during moist and rainy days, had not been properly dried. All farms supplied by these plants subsequently produced some pigs with nephropathy and growth depression, but after changing the certain suspected feedstuffs, the problems with poor growth of pigs disappeared. As a whole, the frequency and duration of the observed nephropathy in different batches of slaughtered pigs varied significantly (from 1%–2% up to 80%–90% frequency) and have depended on the duration of feeding of various suspected feedstuffs stored for a long time in poor conditions and at high humidity (Figure 1). At the same time, the feed contamination levels of FB_1_ (mean 5564.1 ± 584.4 ng/g for year 2006 or 3254 ± 480 ng/g for year 2007 in Bulgarian and 5289 ± 1034 ng/g for year 2007 or 5021 ± 844 ng/g for year 2008 in South African farms with MPN) and PA (mean 838.6 ± 223.9 ng/g for year 2006 or 904.9 ± 86.5 for year 2007 in Bulgarian and 251 ± 69 ng/g for year 2008 in South African farms with MPN) were comparatively high. Moreover, the percentage of positive samples for the same mycotoxins was subsequently high: above 80% in both cases. Some other nephrotoxic mycotoxins such as CIT or even unknown nephrotoxic metabolite (UM) were also found in the same feed from both countries [1,2].

It becomes more and more clear that the overall concentration of OTA in feed samples found in farms with MPN in Bulgaria and South Africa were substantially lower than the levels 1–2 mg/kg (ppm) required to reproduce MPN/MCN of severity similar to that observed in spontaneous cases [1,2,3,4,6,65,66]. It seems, therefore, that the MPN/MCN in Bulgaria and South Africa may have a multitoxin or multifactor etiology, because it cannot be explained by the concentration of OTA alone. 

The mixture of mycotoxins contaminated various foods/feedstuffs in the field would have at least an additive, if not synergistic toxic effect. The presence of multiple mycotoxins in various feedstuffs or foods presents new concerns since toxicological information on the effects of simultaneous exposure is still very limited. Although, the effect of multiple toxins cannot be easily defined, certain *in vitro* or *in vivo* studies can help us predicting the outcome [22,23,54,55,67,68,69].

Our recent experimental work confirmed multi-mycotoxin nature of this nephropathy. Some recent experiments clearly showed that the toxicity of various strains of the same *Aspergillus ochraceus* group is completely different, depending on their capacity to produce both mycotoxins: OTA and PA. A potent synergistic effect was found between OTA and PA, mycotoxins produced by the same ochratoxinogenic fungi, when the same mycotoxins were given simultaneously to pigs and chickens. We were able to reproduce porcine- as well as chicken nephropathies using a moldy diet containing very low levels of OTA (180–300 µg/kg) in combination with PA [54,55,56,70,71,72].

The production of multiple toxins by a single organism, such as *Aspergillus ochraceus* (which produces OTA and PA simultaneously), or by mixture of fungi presents a problem that has not been sufficiently investigated. Such mixtures of toxins may have additional or synergistic effects in farm animals. PA was suspected to be carcinogenic [73,74] and was found to have DNA-attacking ability in the rec assay [75] as well as to induce chromosome aberations [76]. Some data suggest that feed contamination levels of PA itself up to 4000 ppb have little toxicity (mostly hepatotoxicity) and do not adversely affect body or organ weights or biochemical parameters in chickens [77]. Only DNA breaks in mammalian cell lines [78,79] and an inhibition of rat liver glutathione S-transferase activity in crude extracts *in vitro* [80] as well as PA-induced hepatobiliary excretory dysfunction [81] and liver hemorrhages [82] have been found so far. The more potent toxicity of OTA when it is administered together with PA in all mentioned above studies might be due to synergistic toxic effects between both mycotoxins, as has been reported in mice [83,84], poultry [55,56,71,72,85] or pigs [54]. The OTA-producers used in the mentioned above experiments produced both toxins (OTA and PA) simultaneously. In this context, some authors have suggested about the important role of the pancreatic enzyme carboxypeptidase A in the partial detoxification of OTA in the small intestine in rats [86,87]. On the other hand, Parker and collaborators [88] found that PA inhibits carboxypeptidase activity both “*in vitro*” and “*in vivo*”, and such inhibition may significantly impair the partial detoxification of OTA in the intestinal tract and so be partly responsible for the enhanced toxicity of OTA, when it is ingested together with PA. The PA-induced hepatobiliary excretory dysfunction [81] may also result in decreasing the hepatobiliary excretion of OTA. 

Having in mind our recent experimental studies and mycotoxicological investigations of feedstuffs from farms with spontaneous MPN/MCN we reached to the conclusion, that such synergism between OTA and other mycotoxins (as PA, CIT or FB1) under field conditions may be responsible for the spontaneous MPN [3,4,5] and MCN [6] in Bulgaria and South Africa, which can be caused by relatively low contamination levels of OTA in feed [1,2]. This could explain why the low levels of OTA in Bulgarian feedstuffs for pigs [3,5], chickens [6] or humans [62,89,90] have such high toxic effect on kidneys, when received by spontaneous moldy feed. In the synergism between both mycotoxins, the concentration of OTA in feeding forages is probably more important than that of PA, because the higher contamination levels of PA (60,000 ppb PA in combination with 1000 ppb OTA) [91] have not been able to produce more significant toxic effects. These findings clearly suggest that the increase in OTA-toxicity is probably due to impaired detoxification of OTA by PA.

In a recent study Stoev and collaborators [54] have induced macroscopic kidney damage at the end of the 3-month period in pigs fed on a moldy diet containing such low levels of OTA as 180 ppb in combination with PA, but the microscopic lesions in kidneys were observed at that time in pigs fed on diet containing only 90 ppb OTA in combination with PA. The observed changes were similar to those caused in pigs at the end of the 4-month period of exposure to higher levels (markedly above 200 ppb) of pure OTA in feed [10]. It is therefore important to investigate the effect of combined administration of OTA and other mycotoxins, produced by the same ochratoxinogenic fungi as occur in the field.

It is also found that when OTA and PA are administered together, much lower concentrations of OTA (about 100 ppb) are enough for a significant toxic effect in chickens, expressed by degenerative changes in internal organs, by a depletion of cells in lymphoid tissue and a decrease in lymphoid organ’s weight as well by known changes in various biochemical parameters [85]. Moreover, degenerative and weight changes in kidneys, liver and lymphoid organs as well as immunosuppression were seen recently in chickens at only 0.2 or 0.3 ppm OTA in combination with PA [55,56,70,71,72], whereas such changes had been seen in chickens exposed to significantly higher levels of pure OTA (about 4 ppm) in feed [92,93,94,95]. According to other authors [91], a similar but less pronounced synergistic toxic effect has been observed at higher contamination levels of OTA (1000 ppb). The mentioned above low experimental levels of OTA correspond very well to the feed levels of OTA found in spontaneous cases of MCN in Bulgaria (0.09–0.31 ppm), which suggests its possible multicausal nature.

It is now clear that all difference between classic Danish porcine/chicken nephropathy and Bulgarian nephropathy in pigs (e.g., enlargement of renal lymph nodes, neoplastic changes, significantly enlarged and marbled or pale appearance of kidneys as well as cystic or stronger fibrotic changes in cortex of the kidneys) as described in the Veterinary record’s paper [3] and chickens (e.g., nervous symptoms, vascular and edematous changes in various internal organs and the brain, and subcutaneous or liver and kidney hemorrhages in addition to known degenerative changes in the kidneys, liver and lymphoid organs) as described in Veterinary Research’s paper [6] are probably a result of the effects of other nephrotoxic metabolites, such as PA or FB1, in addition to the toxic effect of OTA or may be attributed to synergistic effects between OTA and other mycotoxins, and the increase in OTA-toxicity [1,23]. Such damages were not observed in nephropathy provoked by pure OTA, given at doses and for periods of exposure similar to those in the field [10,96,97] or associated with Danish MPN [65,98].

A similar synergistic effect between OTA, CIT and FB1, has been seen also in some “*in vivo*” or “*in vitro*” studies. OTA and FB1 were found to induce “*in vitro*” and “*in vivo*” degenerative and apoptotic changes in rat kidney [99,100]. The synergistic effect between OTA and FB1, which was seen “*in vitro*” [67,101], is in line with some “*in vivo*” data [68,102]. Moreover, the DNA damage provoked by the combined treatment with OTA and FB1, measured either by the standard comet assay or Fpg-modified comet assay, showed a synergistic increase in kidney cells “*in vivo*” as indicated by the tail length, tail intensity and OTM (olive tail moment), even at doses that correspond to the daily human exposure in Europe [103]. In the same experiment, the tail intensity and OTM of the kidneys cells of rats receiving combined treatment of OTA and FB1 was much higher than would be a simple sum of values caused by the respective doses of either mycotoxin alone.

A similar report for a kind of synergistic effect between some of the target mycotoxins involved in MCN/MPN was made by Lillehoj and Ceigler [104], who give an example where PA and CIT were innocuous when administered alone, but were 100% lethal when given in combination.

In addition, a synergistic effect has been seen between OTA and CIT in the suppression of Concavalin A-induced proliferation of porcine lymphocyte [105]. A similar synergistic effect between these mycotoxins has been found also in “*in vivo*” studies with poultry, rodent and dogs [106] as well as simultaneously in both “*in vivo*” and “*in vitro*” studies as the co-treatment with OTA and CIT has been observed to increase the major adduct formed by OTA [107]. It was found that simultaneous administration of OTA and CIT also enhanced the incidence of renal cell tumours in mice [108]. 

The synergistic interaction in cytotoxic effect between FB1 and OTA may be related to the ability of both toxins to impair protein synthesis and to increase lipid peroxidation producing reactive oxygen species [109,110]. Because these toxins are increasing reactive oxygen species production, the feeding animals with diet containing antioxidants may be a good preventive measure [101,111,112].

We should have in mind that some rare and slightly nephrotoxic mycotoxins as xanthomegnin, cyclopiazonic acid, erythroskyrin and rubratoxin B may also have an additional synergistic or additive nephrotoxic effects in addition to already mentioned mycotoxins as the same were found in many of the feed samples from farms with MPN in Bulgaria and South Africa [1,2]. 

Some recent investigations on the cytotoxic effect of different combinations of target mycotoxins involved in MPN/MCN as OTA, PA, CIT and FB1 on human peripheral blood mononuclear cells measured by MTT (Methylthiazol Tetrazolium) assay revealed additive or synergistic effects of the following mycotoxins: OTA, CIT and FB1 as compared to any single mycotoxin [113]. In the same experiment, there was not observed “*in vitro*” synergistic effect between OTA and PA, which can be explained by the specific mechanism of “*in vivo*” synergistic effect of both mycotoxins and MTT assay being inappropriate for cytotoxic evaluation of PA as the same mycotoxin increases metabolic activity of cells. It is important to recognize that “*in vitro*” experiments can mainly demonstrate direct synergistic effects of mycotoxins, but only “*in vivo*” studies can clearly show the real interaction between mycotoxins in addition to their absorption, distribution, bioavailability, metabolism and excretion. Obviously, it is already of great importance to determine whether feeding such mycotoxins contaminated moldy feed to pigs/chicks could reproduce the same functional and morphological changes in kidneys observed in spontaneous porcine/chicken nephropathy at contamination levels and periods corresponding to the levels and periods of exposure to feed naturally contaminated with the same combination of mycotoxins. In addition, it will be helpful to establish, whether the same combination of mycotoxins has a real synergistic effect “*in vivo*” as this one between PA and OTA [54,55].

FB1 [114,115] and PA [73,74] were also found to be carcinogenic mycotoxins and may interact in this dimension with OTA, which is also proven carcinogen [76,116,117,118]. Moreover, FB1 was found to have a pronounced nephrotoxic effect on animal kidneys [61,119], which can be additive to nephrotoxic effect of OTA. On the other hand, FB_1_ also decreases myocardial contractility in pigs. This is thought to be mediated by increased sphingosine inhibiting the L-type calcium channels in the myocardium [120]. In such a way, left sided heart failure results in pulmonary edema, which is the main distinguishing feature of FB_1_ toxicity in pigs [102,121].

It has to be emphasized that FB1 may also contribute to the immunosuppressive effect of OTA and the increase in secondary bacterial infections observed in pigs with spontaneous MPN [57], because of an increase in intestinal colonization by pathogenic *E*. *coli* [58].

In addition to various synergistic effects between target mycotoxins, we must have in mind the specific characteristics of each mycotoxin as its transfer to the milk or through the placenta to the fetus and the possible effect on the animal progeny [122,123,124] as well as its bioavailability and degree of binding with plasma proteins [117] in order to establish its potential risk for animals or humans. 

Having in mind the potent synergistic effects between OTA and PA or CIT [54,83,105] as well as between OTA and FB1 [67,101], simultaneous exposure to those mycotoxins might be of significant importance and could be crucial for development of chronic renal failure observed in MPN in Bulgaria and South Africa or Balkan Endemic Nephropathy (BEN), especially after long-term ingestion of the same mycotoxins. Therefore, it is already a time to understand, whether such a combination of mycotoxins is occurring in food and feed and what the ranges and contamination levels are. It is a pity that there is only scarce information about the contamination levels of PA and FB1 in foods or feedstuffs from MPN- and BEN-endemic areas, because extensive studies have not been performed so far. It has only been found that FB1 and OTA co-occurred in maize in Croatia [125,126,127] as the mean levels of FB1 (459.8 μg/kg) found in the last study [127] are not so low. High contamination levels of OTA and FB1 (up to 40 mg/kg) have been also found in some pig feedstuffs [128] and were reported to provoke the death of the same pigs as pathological picture revealed pathological signs of both toxins, e.g., pulmonary edema, liver and kidney lesions.

## 4. The Joint Mycotoxin Interaction and Its Relationship to Balkan Endemic Nephropathy

It is important to mention that the low feed- and serum levels of OTA in MPN-affected farms [1] are also very similar and comparable to those in food and blood of humans with BEN, widely ranged in Bulgaria and other Balkan countries [89,129,130]. The similar toxicological (Table 2, Table 3) or pathomorphological (Table 4) findings strongly suggest a possible common etiology in both diseases—BEN and MPN [1,23,90].

Worryingly, OTA and CIT [131] in addition to OTA, FB_1_ and PA [1] are often present in human, but not only in animal foods/feedstuffs in Bulgaria originated from BEN-endemic areas or from farms with MPN/MCN. Moreover, the feed levels of the same mycotoxins are not so low in order to be neglected and the amount of CIT has been often ten times higher in bean or maize from BEN-affected families compared to the non-affected ones [131]. Some recent investigations also confirmed the implication of OTA and CIT in BEN, because DNA adducts related to OTA [132] and CIT [133] were found in human kidney tissues from BEN-endemic areas, and both mycotoxins were present in high percentage of investigated food/wheat samples as well as in the urine of families with BEN [62,132].

**Table 2:** A content of ochratoxin A (OTA) in various food and feed samples from endemic and nonendemic of Balkan endemic nephropathy (BEN) areas as well from farms with MPN and without MPN in Bulgaria.

Areas (farms)	Year	Food or feed	Number of samples	Number of positives	% of positive	Range/mean (µg/kg)	Reference
Endemic of BEN	1986	Beans	34	13	38.2	25–200	[129]
		Maize	34	14	41.7	25–250	
	1989	Beans	30	11	36.6	25–240	
		Maize	32	14	43.7	25–900	
	1990	Beans	25	10	40.0	85–260	
		Maize	25	11	44.0	25–890	
Nonendemic	1986	Beans	24	2	8.3	20–150	[129]
		Maize	24	2	8.3	20–180	
	1989	Beans	25	2	8.0	25–200	
		Maize	25	2	8.0	10–230	
	1990	Beans	40	2	5.0	10–220	
		Maize	40	2	5.0	20–235	
Farms with MPN	1993	feed	7	7	100	38–552	[3]
	1994	feed	10	10	100	42–427	
	2006	feed	25	25	100	188 ± 27	[1]
	2007	feed	25	25	100	376 ± 63	
Farms without MPN	1993	feed	5	-	-	-	[3]
	2006	feed	25	-	-	-	[1]

**Table 3:** A content of OTA in various blood samples from the humans/pigs in endemic and nonendemic of MPN/BEN areas in Bulgaria.

Areas (farms)	Year (season)	Number of samples	Number of positives	% of positive	Mean ± SEM (µg/kg)	References
Endemic of BEN	1986	30	7	23.3	20.0 ± 2.0	[130]
	1989	24	7	29.2	27.2 ± 11.9	
	1990	20	5	25.0	25.0 ± 10.6	
Nonendemic	1986	52	4	7.7	10.0	[130]
	1989	30	2	6.6	0 < 2	
	1990	30	2	6.6	8.0	
Farms with MPN	autumn 1993	25	16	64	4.8 ± 0.9	[5]
	spring 1994	25	25	100	60.9 ± 9.2	
	autumn 1994	25	12	48	21.9 ± 14.2	
	2006	10	8	80	28.8 ± 25.1	[1]
	2007	10	9	90	6.3 ± 4.9	
Farms without MPN	1993	5	-	-	-	[5]

**Table 4:** The main morphological characteristics of the renal lesions in Bulgarian MPN and BEN [3,4,90].

Parameters	Bulgarian MPN	BEN
Size of kidneys:		
✓ in early stages	enlarged	no information
✓ in later stages	reduced towards former stages	strongly reduced
Degenerative changes mainly in proximal tubules	+ (mostly in early stages)	+ (mostly in early stages)
Granular and hyalin casts or necrotic debris in the tubules	+ (mostly in early stages)	+ (mostly in early stages)
Dilated atrophic tubules (retention cysts)	+ (in later stages)	+ (in later stages)
Interstitial fibrosis and hyalinization/sclerosis of glomeruli	+ (in later stages)	+ (in later stages)
Mononuclear (inflammatory) cell infiltration	+ (moderate)	+ (slight)
Dilated lymphatics (lymphatic cysts)	+ (mostly in later stages)	+ (mostly in later stages)
Vascular lesions	+	+
Neoplastic changes	+ (benign tumour in kidneys)	+ (malignant tumours in ureters/pelvis)
Ultrastructural damages in proximal tubules:		
✓ Reduced brush border in high and density	+	+
✓ Diminution or disappearance of mitochondrial cristae	+	+
✓ Swollen and distorted mitochondria	+	+
✓ Myelin-like figures and lipid droplets in the mitochondria	+	+
✓ Electron dense formations in the nuclei and mitochondria	+	+
✓ Large number apical vesicles	+	+
✓ Lost membrane integrity of cell organelles	+	+
✓ Increased number of phagolysosomes	+	+
✓ Thickening of basement tubular membranes	+ (in later stages)	+ (in later stages)

Furthermore, the comparative ten-year follow-up study of OTA exposure in a BEN endemic village in Croatia clearly showed higher frequency of OTA-positive food and serum samples than in control village [133]. OTA was also more frequent and at higher levels in blood samples from patients with BEN and urinary tract tumours compared to non-affected ones [133]. However, one important factor must be emphasized, when comparing MPN and BEN: MPN is developed in the course of a few months, whereas BEN takes many years to reach a state which makes clinical diagnosis possible, which probably is due to a longer exposure of human to OTA in smaller doses. We must also have in mind that a slow excretion of OTA supposes a tendency to an increase in toxin levels in the serum as well as assumes a very constant rate of OTA in human blood for a long period of time, even after repeated exposure to very low concentrations [134]. In such way humans are more influenced by the dose and less influenced by the frequency, duration and quantity (if the quantity of the toxin ingested is comparatively low) of OTA exposure than other species, because in the humans OTA has a very constant and continuous effect on the proximal tubules even in a little amount, as well because of the very low unbound (free) fraction of OTA in the blood. It could suppose that only the free fraction of OTA in the macro-organism has a toxic effect on the tissues. Such a continuous persistence of OTA and especially its free fraction in human blood and its slow excretion via kidneys could be partly responsible for the slow progression of BEN. Therefore, the consumption of food containing very low concentrations of OTA over a long period of time may become toxicologically significant [1,23,90,132]. 

The pathomorphological changes in kidneys in spontaneous MPN in Bulgaria, including fibrotic changes and contraction of kidneys in later stages, resemble much more to those in BEN in humans, than in Danish MPN [23,90], which can also support the possible multimycotoxic nature of both diseases BEN and MPN in the Balkan countries. Furthermore, there are many other striking similarities between BEN in humans and Bulgarian MPN as the low food/feed or blood concentrations of OTA (Table 2, Table 3), neoplasia in kidneys (pigs) or urinary tract (humans), retention tubular cyst formations, vascular damages, electron dense formations or myelin-like figures in mitochondria of epithelial cells (Table 4). The same damages have not been seen in classical MPN as described in Scandinavian countries or elsewhere. All these discrepancies between Bulgarian MPN and classical MPN, in addition to the similarities between Bulgarian MPN and BEN, could be due to the interaction between OTA and other mycotoxins, which needs to be further proved. Our arguments can be also supported by some recent experiments, in which we found electron-dense formations in the nuclei and myelin-like figures in the epithelial cells of proximal tubules of kidneys of pigs exposed to very low contamination levels of OTA together with PA. The same damages resemble much more to those in spontaneous MPN or BEN in the Balkans and are unusual for classical Danish MPN [54] (Table 4).

Having in mind all these similarities between both nephropathies, it becomes clearer why MPN is considered to be the best model for BEN in humans [90]. 

## 5. The Implication of Some Fungi and New Fungal Metabolites in Etiology of MPN/MCN/BEN

The absence of conventional ochratoxinogenic fungi (*P*. *verrucosum*) as significant contaminants in Bulgarian and South African feed samples demonstrates that such molds are not common in these countries [1,2]. Furthermore, to ascribe the source of OTA to the *A*. *ochraceus* group of fungi is unwise because ochratoxinogenic forms are isolated so rarely [1,2,3,135].

Some mycological investigations of OTA-contaminated feedstuffs in Bulgaria revealed the common presence of *P*. *aurantiogriseum* complex [1,2,3], which is a potent producer of PA [136]. PA is produced by numerous species of *Penicillium* (especially *P*. *aurantiogriseum*) and *Aspergillus* at temperatures ranging from 4 °C to 30 °C, with the maximum rate of production occurring at about 25 °C [137]. The production of PA decreased sharply at low oxygen concentrations, while fungal growth was not noticeably influenced [49]. PA progressively forms complexes with compounds containing -SH radicals [138,139]. The rate of toxin coupling with -SH radicals increases with pH as well as in high temperature [139]. The resulting complexes are much less toxic than the uncoupled molecules, resulting in actual detoxification. Therefore, this toxin usually accumulates at relatively low temperatures during the winter at which detoxification is more reduced than toxin production [137]. That could explain why Bulgarian nephropathy in pigs/chickens was usually observed during the spring (after the winter) [3].

Recently, it has been found that administration of *P*. *polonicum* extract (not containing OTA) to rats can provoke profound and persistent histopathological damages, including apoptic and karyomegalic or mitotic changes in the nuclei of tubular epithelium in kidneys of rats and DNA-adducts formation [140]. The same *P*. *polonicum* strain, which is a common food/feed spoilage mold in warm temperate latitudes, has been found as a frequent contaminant in Bulgarian feedstuffs, suspected of causing spontaneous porcine nephropathy [1,3,135]. It can be supposed that *P*. *polonicum* extract given to rats probably contained PA. It is known that the *P*. *aurantiogriseum* group (including *P*. *polonicum* strain) is usually a potent producer of PA, which can also provoke DNA breaks in mammalian cell lines as have been reported previously [78]. Recently, it was found that the main source of PA in Bulgarian feedstuffs was different from the source of OTA [1]. In addition, the same changes (apoptosis and karyomegaly in tubular epithelium), provoked by *P*. *polonicum* extract, could be induced by another not yet identified mycotoxin (unknown mycotoxin—UM), which needs to be further proven. 

It seems that the same not yet chemically identified mycotoxin (UM), found to contaminate almost all feed samples from farms with MPN, could be partly responsible for the known nephrotoxic damages in Bulgarian animal/human nephropathy. Our recent study [141] revealed a potent cytotoxicity of the same purified UM. Furthermore, the quiet apoptosis induced by the same UM, which can be produced mainly by *P*. *polonicum*, could be partly responsible for the cryptic and clinically-silent onset of renal atrophy in the idiopathic BEN in humans [142]. On the other hand, apoptotic changes provoked by UM could couple with apoptotic changes, which can be provoked by OTA. It has been reported a strong potency of OTA to induce apoptosis and DNA-adducts *in vitro* [143,144] and *in vivo* [145,146,147,148,149,150], and that mycotoxin was considered to be responsible for the DNA-adducts in the urinary tract tumors of patients with BEN [62,151]. Therefore, the extent to which OTA may interact with other components of commercial chicken/pig rations or human food compounded from agricultural produce may also influence the significance of the relatively lower doses of OTA that commercial chickens [6], pigs [3,4,5] or humans [90] may encounter in some feed/food. 

It is already of great importance to pay more attention to the high incidences of *Penicillium* spp. (especially *P*. *polonicum*) responsible for the high contamination levels of this UM in animal and human feed/food in Bulgaria. It is also essential to study other potential biological effects of the UM on mammalian cells in further “*in vivo*” or “*in vitro*” studies. Moreover, it is also important to perform some further investigations, including nuclear magnetic resonance, in order to clarify the chemical structure of this UM.

It is crucial to mention, that besides *P*. *polonicum*, some other *Penicillium* fungi such as *P*. *aurantiogriseum* and *P*. *commune* were also found to be nephrotoxic to rats [152] or to kidney tubule cells in tissue culture [153]. These fungi were isolated from maize collected from BEN-endemic areas of former Yugoslavia and the authors conclude that the same fungi produce biologically active fraction or secondary metabolite (unknown mycotoxin), which could be a possible factor in the etiology of BEN. The toxicity of this compound is a little similar to that of OTA and the target place was concluded to be the P3 segment of proximal tubules of kidneys. Such fungi and UM were also isolated from MPN-endemic areas in Bulgaria [1,3]. Recently, we managed to isolate such a substance (UM) from the same *Penicillium* fungi, isolated from MPN-endemic areas in Bulgaria. The same substance was recently purified and studied for possible cytotoxic effect on human lymphocytes in comparison to other mycotoxins such as OTA and T-2 toxin and its toxicity at low concentrations (0.15, 0.31 and 0.63 µg/mL) was found to be lower than toxicity of OTA, but a little similar to that of T-2 toxin [141].

## 6. The Significance of the Masked Target Mycotoxins and Their Binding to Some Serum Macromolecules in the Etiology of MPN/MCN/BEN

It is well known that plants can metabolize xenobiotic compounds including mycotoxins as part of their defence against pathogens, whereas animals can also actively eliminate (excrete) mycotoxins via the kidney and liver. The definition of masked mycotoxins implies that the analysis of the mycotoxins content of samples leads to underestimation of the real levels of mycotoxins and their derivatives. Mycotoxin derivatives that are undetectable by conventional analytical techniques because their structure has been changed in the plant are designated as masked mycotoxins [154]. The chemical transformations that generate masked mycotoxins are catalyzed by some plant enzymes involved in detoxification processes [155]. 

Masked mycotoxins could represent large amount of total mycotoxins. It is supposed that the ingested masked mycotoxins could give back the native mycotoxins after microbial and/or animal metabolisms [155]. Unfortunately, the masked mycotoxins are usually not considered in the measurement of native toxins in food, but the same could represent a large amount of total toxins. Having in mind the circumstance that masked toxins could be transformed in native toxins after ingestion, it can be supposed that the found levels of target mycotoxins in foods/feedstuffs are often actually higher, because masked mycotoxins have not been measured. Therefore, masked toxins could contribute to the observed difference between the dose of native mycotoxins ingested and their actual contamination levels. 

The group of masked mycotoxins comprises both extractable conjugated and bound (non-extractable) varieties. Bound mycotoxins are covalently or non-covalently attached to polymeric carbohydrate or protein matrices [156]. Extractable conjugated mycotoxins can be detected by appropriate analytical methods when their structure is known and analytical standards are available. Bound mycotoxins, however, are not directly accessible and have to be liberated from the matrix by chemical or enzymatic treatment prior to chemical analysis [155]. Bound mycotoxins may be regarded as detoxified as long as they cannot be released from the matrix during food processing or in the digestive system of animals/humans. Food processing may also chemically alter mycotoxins [7], but usually most of the food-processing compounds are less toxic than their precursors. The fermentation processes may transform mycotoxins into products that are also not detected by analytical methods conventionally used for mycotoxin monitoring [155]. 

Some conventional analytical methods, such as ELISA, might respond to masked forms, whereas this is unlikely for HPLC-based methods. All analytical methods for parent mycotoxins are potentially also suitable for their conjugated forms. However, it should be known that only soluble analytes are directly accessible for analysis. Bound forms cannot be detected without sample treatment that converts them into soluble forms, while soluble conjugated forms can be determined after extraction with typical solvents used in mycotoxin determination [155]. Transformation of masked mycotoxins into parent molecules usually involves hydrolysis [157], the type of which has to be carefully selected. Unfortunately, no current single hydrolysis method is applicable to all masked mycotoxins. Sometimes, masked mycotoxins might be less toxic than their parent compounds, if the hydrolysis of glucosides during digestion is incomplete. However, masked mycotoxins might also be more toxic than their parent compounds, e.g., when they are more bioavailable [155].

Several reports have shown the presence of bound fumonisins in food, which can be determined only after application of a hydrolysis step [158,159,160]. It has been also observed that after performing alkaline hydrolysis of contaminated corn products (e.g., extruded products such as corn flakes) the amount of fumonisins released was often higher than the expected one. 

The plant metabolism of OTA was investigated using various cell suspension cultures of wheat and maize incubated with ^14^C-OTA [161]. It was found, that in addition to OTα, the main metabolites isolated were (4*R*)- and (4*S*)-4-hydroxy-OTA. In addition, β-glucosides of both isomers have been found in large quantities. OTα is considered to be a non-toxic metabolite, whereas hydroxy-OTA is found to have a potent toxicity similar to this of OTA itself, including immunosuppressive effect [161]. However, for OTA, no further toxicological studies on its plant metabolites were performed. 

Some experiments performed on rats have shown that OTA can be hydrolysed into OTα, partly microbially in the caecum and large intestine and partly in the cells in the duodenum and jejunum. OTA is not however hydrolysed “*in vitro*” by kidney and liver homogenates. A small part of OTA can be hydrolyzed by the microsomal fraction within the liver. Stormer and Pederson [162] showed that liver microsomes from humans, pigs and rats are capable of metabolizing OTA in the presence of NADPH to (4*R*) and (4*S*) hydroxy OTA. *In vivo* studies revealed that 1% to 1.5% of orally ingested OTA was excreted in the urine as (4R) hydroxy OTA and 25 to 27% as OTα, whereas in the faeces 9% was excreted as OTα and 12% as OTA [163]. Other OTA-metabolites are excreted in a different way by male and female (rat/pig/human). However, we should have in mind that some of them, such as OTHQ (ochratoxin hydroquinone) are related to carcinogenicity [63,164,165,166]. It has been also hypothetised that the interaction between OTA and CIT is due to the autooxidation of CIT inducing formation of OTHQ [69].

The relative quantity of OTA eliminated via liver and kidneys of animals/humans and the difference in OTA persistence in plasma depends however on several factors, including the animal species, the dosing route and dose, the enterohepatic recirculation, and OTA’s binding to serum macromolecules [132,167]. The fraction of OTA that is bound to serum macromolecules constitutes a mobile reserve of the toxin that can be released to the tissue over a long period of time. Therefore, the protein bound to OTA could delay toxin elimination by limiting its transfer from the blood stream to the hepatic and renal cells. It has to be emphasized that OTA has been found to possess nearly 10^6^ times higher affinity to the not yet unidentified serum macromolecule with a lower molecular weight (about 20,000) than to serum albumin [168,169]. OTA is presumed to have a very long plasma half-life in humans because the studies have revealed that ochratoxin A has a very high affinity to this macromolecule in human serum [170]. The plasma half-life of OTA depends strongly from its bound to such serum macromolecules and ranges from 4 h in chickens to 510–840 h in monkeys. However, the second-longest half-life 72–120 h is found in pigs [171]. 

On the other hand, Macanovic and his collaborators have observed intense deposits of IgG and complement component C_3_ on the tubular basement membrane in the early stage of the BEN [172]. The increased deposits of IgM and C_3_ in glomerular capillary walls are also established in some cases of BEN [173]. Some other authors have reported for a presence of antibodies in the human sera of patients living in endemic areas of ex-Yugoslavia [174]. It is found that 58.49% of patients suffering from BEN in Bulgaria have cellular-determined immunity from soluble antigens in renal epithelial cells [175]. All these findings would indicate that BEN is an immune-complex-type nephritis. Therefore, OTA, in addition to the nephrotoxic property may demonstrate an antigenic property when is conjugated to a protein, *i*.*e*., act as a hapten. Thus, OTA-albumin complex may hypothetically cause production of specific antibodies and a repeated exposure may cause formation of antigen-antibody complexes on the tubular basement membranes of the proximal tubules [98,176]. 

Some other authors assume that OTA bound to macromolecules of such low molecular weight as a 20,000 may pass the glomerular membranes and may be trapped by pinocytosis in the proximal tubular cells, accumulating in them and possibly exerting known nephrotoxic effects [177]. The saturation of these macromolecules is reached with such a low concentration of OTA as 10–20 ng/mL serum [177], frequently encountered in endemic areas. Enterohepatic circulation of OTA in the human body could maintain the saturation of the specific binding macromolecules for a long time [90,132]. 

On the other hand, it may be supposed that only a free fraction of OTA may pass the gromerular membranes and may be reabsorbed in the proximal tubules, like enterohepatic circulation. Such recycling could be responsible for kidney lesions and moreover contributes to a long half-life of OTA in various animals and human [90]. On the other hand, it has been shown that CIT may decrease OTA excretion in rat [107]. 

Unfortunately, at present there are no sufficient toxicokinetic and toxicodynamic studies available to reveal possible hazard or risk assessment of masked mycotoxins in comparison with the parent mycotoxins. Therefore, it is impossible to perform a proper risk assessment for masked mycotoxins in food, due to the lack of data on exposure and toxic properties. There is a defined need for more toxicological studies comparing the masked mycotoxin with its parent.

The recognition of the toxicological relevance of masked mycotoxins in food commodities is a new challenge which should be considered by the respective regulatory bodies, food manufacturers and monitoring authorities in order to protect the health of consumers. The same masked mycotoxins and their target combinations may significantly add to the development of porcine/chick or human nephropathy, but that need to be carefully investigated via some well-designed studies. 

## 7. Conclusions

It seems that in a diverse human or animal diet, exposure to multiple mycotoxins at a low concentration and at an intermittent rate over long period of time may cause toxic damage to the kidneys. It is therefore of great importance to investigate the real toxic effect of combined administration of OTA and other mycotoxins, as it is occurred in the field. 

Mycotoxins are natural contaminants, and therefore, human exposure cannot be completely prevented. Obviously, the development of national programs for the monitoring, prevention and control of mycotoxin contamination based on the assessment of the situation in each individual country is not sufficient these days. The factors which are compromising the quality of the products of the commodity system, and leading to the production of molds and mycotoxins, should be evaluated by the implementation of carefully designed surveillance studies and modern internationally recognized biomonitoring methods measuring the exposure to mycotoxins of individuals. However, it has to be realized that only an integrated approach to food safety, which includes systematic identification and assessment of hazards in foods/feedstuffs and various means to control them, could resolve the current problems in this field. Effective enforcement of food safety laws and regulations in addition to surveillance control is also required to reduce the number of food-borne diseases as well as to enhance foods/feedstuffs security. Furthermore, a harmonization of various national standards in regards to various mycotoxins, including masked mycotoxins, and their joint toxic effects and interaction is also necessary for protecting the consumer and ensuring a global safety of various kinds of foods/feedstuffs produced in different countries from all over the world.
